# Genomic context analysis reveals dense interaction network between vertebrate ultraconserved non-coding elements

**DOI:** 10.1093/bioinformatics/bts400

**Published:** 2012-09-03

**Authors:** Slavica Dimitrieva, Philipp Bucher

**Affiliations:** ^1^Swiss Institute for Experimental Cancer Research (ISREC), School of Life Sciences, Swiss Federal Institute of Technology (EPFL); ^2^Swiss Institute of Bioinformatics (SIB), CH-1015 Lausanne, Switzerland

## Abstract

**Motivation:** Genomic context analysis, also known as phylogenetic profiling, is widely used to infer functional interactions between proteins but rarely applied to non-coding *cis-*regulatory DNA elements. We were wondering whether this approach could provide insights about utlraconserved non-coding elements (UCNEs). These elements are organized as large clusters, so-called gene regulatory blocks (GRBs) around key developmental genes. Their molecular functions and the reasons for their high degree of conservation remain enigmatic.

**Results:** In a special setting of genomic context analysis, we analyzed the fate of GRBs after a whole-genome duplication event in five fish genomes. We found that in most cases all UCNEs were retained together as a single block, whereas the corresponding target genes were often retained in two copies, one completely devoid of UCNEs. This ‘winner-takes-all’ pattern suggests that UCNEs of a GRB function in a highly cooperative manner. We propose that the multitude of interactions between UCNEs is the reason for their extreme sequence conservation.

**Supplementary information:** Supplementary data are available at *Bioinformatics* online and at http://ccg.vital-it.ch/ucne/

## 1 INTRODUCTION

Genomic context analysis (Huynen *et al.*, 2000) is a comparative evolutionary approach to infer interactions between protein-coding genes. The STRING database (Szklarczyk *et al.*, 2011) distinguishes three types of evolutionary evidence for interactions: co-occurrence in the same species, co-localization in the same chromosome region and gene fusion. Co-occurrence analysis is the most widely applicable variant and has been termed phylogenetic profiling (Pellegrini *et al.*, 1999). The co-localization or neighborhood criterion is primarily used in prokaryotes where genes participating in the same pathway are often arranged in a single operon. Gene fusion is a less frequent event, but probably constitutes the strongest type of evolutionary evidence that two proteins encoded by separate genes in some genomes physically interact with each other.

In principle, genomic context analysis should be applicable to non-coding sequences as well. The neighborhood criterion is ideally suited to infer interactions involving *cis*-acting regulatory elements as such elements by definition have to reside on the same chromosome with their interaction partners. Nevertheless, with a notable exception discussed further below, genomic context analysis has rarely or never been applied to non-coding sequences. In this work we present an application of this method to vertebrate ultraconserved non-coding sequence elements, henceforth referred to as UCNEs.

Vertebrate UCNEs are the most conserved sequences that exist in nature. The term was originally coined by Bejerano *et al.* who defined ultraconserved elements as sequence regions that are at least 200-bp long and 100% identical between human, mouse and rat (2004). Somewhat different definitions have been applied by other groups (see Elgar and Vavouri, 2008). In a previous work, we defined UCNEs as non-coding elements that are at least 200-bp long and have evolved at a substitution rate of *<*0.01% per million years (Retelska *et al.*, 2007).

The reasons for ultraconservation remain enigmatic. No molecular mechanism is known that would require such a high degree of conservation. It is broadly accepted that a majority of UCNEs act as tissue-specific enhancers (Woolfe *et al.*, 2005). A few may function as splicing regulators (Lareau *et al.*, 2007) or as non-coding RNAs (Calin *et al.*, 2007). Surprisingly, genetically altered mice lacking particular UCNEs were found to be viable with no detectable phenotype (Ahituv *et al.*, 2007). It could be argued that the phenotypes may be too mild to be recognized by human researchers but still severe enough to be removed by purifying selection in nature over many generations.

A striking property of UCNEs is their tendency to occur as large clusters on the genome (Sandelin *et al.*, 2004). Such clusters, also referred to as genomic regulatory blocks (GRBs), may comprise up to 100 UCNEs, span over more than a megabase, and include up to a dozen of genes. Within a GRB, the UCNEs appear to be randomly localized with respect to genes, with approximately equal numbers located in introns and intergenic regions, respectively. The order of individual elements is strictly conserved between distant vertebrate species resulting in perfect synteny of the corresponding genes. It is assumed that GRBs in general have only one target gene. The other genes within the block are called bystander genes.

In an elegant and pioneering study by Kikuta *et al.* (2007) the neighborhood principle of genomic context analysis was used to discriminate between target and bystander genes of GRBs in fish genomes. For most vertebrate species, the neighborhood criterion would be useless because the synteny within GRBs is perfectly conserved. However, the situation is radically different in teleost fishes which have undergone a lineage-specific whole-genome duplication (WGD). After the WGD event, most genes survived in only one copy and the same may be true for gene regulatory elements as well. Within a duplicated GRB, bystander genes may be retained randomly by one or the other copy. However, the UCNEs will always stay with their target genes. The availability of five fish genomes with potentially different gene loss histories after WGD increases the power of the method. In fact, numerous cases of reciprocal gene loss were observed between the lineages leading to zebrafish and *Tetraodon* (Semon and Wolfe, 2007). We should mention in this context that the fate of conserved non-coding elements after WGD was also analyzed to address questions about subfunctionalization (Woolfe and Elgar, 2007).

**Fig. 1.**
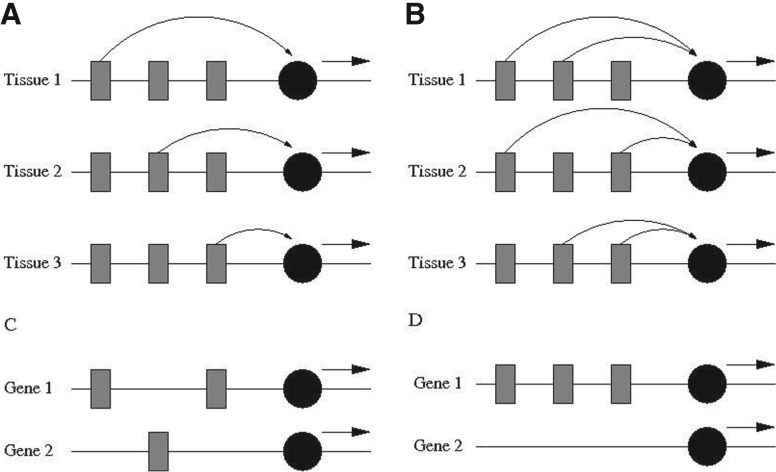
Alternative models of UCNE action and corresponding retention patterns after whole-genome duplication. Grey rectangles represent UCNEs (supposed to be remote control elements), the black circles represent the promoter of the target gene (not supposed to be ultraconserved). (**A**) Stand-alone model: each UCNE drives independently of the other UCNEs the expression of the target gene in one particular tissue. (**B**) Cooperative model: the simultaneous activity of at least two UCNEs is required for target gene expression. Different combinations of UCNEs drive expression in different tissues. (**C**) Reciprocal retention pattern after WGD expected under the stand-alone model: UCNEs get randomly distributed over the two daughter genes. (**D**) ‘Winner-takes-all’ retention pattern expected under the cooperative model. UCNEs need to be retained by the same daughter gene in order to ensure expression in all tissues

In this work, we also exploit the WGD event that has happened in teleosts. However, we address a different question. We are not primarily interested in UCNE–target gene relationships. Our focus is on cooperative interactions between UCNEs within the same block. Specifically, we use genomic context analysis to discriminate between two models of UCNE action, which may be called ‘standalone’ and ‘cooperative’. The standalone model assumes that each UCNE acts independently, whereas the cooperative model assumes that two or more UCNEs jointly drive expression of the target gene in a particular tissue. The two modes of gene regulation are illustrated in Figure 1 with a minimal GRB consisting of three UCNEs. In the standalone mode, each UCNE drives gene expression in one of three different tissues. In the cooperative mode, three different pairwise combinations drive the expression in three different tissues. The fate of UCNEs after WGD could potentially discriminate between these two scenarios. If UCNEs act independently of each other they will be randomly distributed over the daughter genes. If UCNEs cooperate with one another they have to be jointly retained with one daughter gene. We should mention that the discriminatory power of this approach depends on two conditions: (i) the target genes are frequently retained in two copies after WGD and (ii) individual UCNEs are frequently retained in only one copy. Fortunately, these conditions are met, as will become evident from the results presented further below.

The reminder of this article is organized as follows. Section 2 describes the computational protocols at a technical level. In Section 3, we present two largely independent studies. The first part centers on putative target genes that are highly enriched in intronic UCNEs. In the second part, we analyze the fate of the most prominent GRBs after WGD. This part also includes a non-technical description of the computational protocol used to trace-back the individual pieces of duplicated GRBs after potential synteny breaks. Conclusions and perspectives are presented in the last section.

## 2 METHODS

### 2.1 Identification of UCNEs

We scanned whole-genome alignments between human (hg19) and chicken (galGal3) genomes provided by the UCSC Genome Browser (Fujita *et al.*, 2011) in order to extract the human sequence regions where the percentage of sequence identity consistently is ⩾95%. These two reference species were selected for this work because they are well suited to define synteny blocks in view of the evolutionary distance and high quality of genome assembly (Section 2.5.1). The sequence identity was computed in an asymmetric fashion by taking the human genome as a reference and counting the number of conserved bases in the target species in a 61-bp sliding window. According to previous experience (Retelska *et al.*, 2007), this window size offers a good trade-off between spatial resolution and stability of the signal. The number obtained from one window was used to assign a percentage identity value to the base at the center of the window. The numbers from the first and last window were also used to assign missing percentage identity values to the 30 bases at the beginning and at the end of the aligned sequence regions.

Based on the ‘RefSeq Genes’ annotation track for the human genome (hg19) downloaded from the UCSC Genome Browser, we filtered out sequences (or parts of sequences) that overlap with coding regions. After that, we eliminated sequences that were *<*200 bp. The remaining 4386 sequences composed our set of UCNEs (Supplementary Table S1).

Each of these UCNEs was classified as intronic, UCNE within an untranslated region (UTR) or intergenic using the human RefSeq gene annotation.

### 2.2 Identification of UCNE homologs in fish genomes

We analyzed the retention of each UCNE in five fish genomes: *Fugu* (fr2), medaka (oryLat2), stickleback (gasAcu1), *Tetraodon* (tetNig2) and zebrafish (danRer7). We used the program SSEARCH v36.3.5 from the FASTA package (fasta.bioch.virginia.edu/) (Pearson, 1996) to compute the optimal local alignment score between each human UCNE and each conserved fish genomic sequence. For this purpose, we extracted all fish sequences alignable to a human region according to the fish-human pairwise alignments downloaded from the UCSC Genome Browser. SSEARCH was then again used *a posteriori* to compute a base composition-adjusted *E*-value for each aligned region by shuffling the fish sequence 500 times in windows of 20 bp. Aligned fish sequences with *E*-value ⩽ 10^−4^ were accepted as homologs.

### 2.3 Identification of paralogs of UCNEs

A fish homolog of a human UCNE could be a direct ortholog of the UCNE under consideration, or an ortholog of a paralog of the human UCNE. To eliminate paralogs of the latter type, we first compiled a list of paralogs of human UCNEs. This was done by aligning each human UCNE to all human sequences extracted from human–chicken pairwise alignments from UCSC, using SSEARCH exactly as described above. Note that the human paralogs identified in this way may themselves not qualify as UCNEs. Using the human paralog list, we compared each fish homolog of a human UCNE to all human paralogs if there were any. If a better alignment score (lower *E*-value) was obtained with a paralog, then the fish homolog was considered a paralog of the specific human UCNE under consideration.

### 2.4 Analysis of UCNE-enriched genes

#### 2.4.1 Classification of retention patterns of intronic and UTR UCNEs in fish orthologous genes

Our initial set of UCNEs consists of 2220 UCNEs residing within introns and UTRs of 618 human genes. Orthologous genes in the five fish genomes were compiled from Ensembl v64 (Flicek et al., 2011). Genes annotated as ‘*possible orthologs*’ were also included. We discarded the human genes for which either none of the fish orthologous genes retained UCNEs or none of the fish species retained more than one ortholog. In total, we analyzed 204 test cases of gene–UCNE associations. The UCNE retention patterns were then assigned to three groups referred to as ‘winner-takes-all’, ‘concordant retention’ and ‘reciprocal retention’. To this end, we counted the number of UCNEs retained by each gene of a group of orthologs in a fish species. The orthologous gene with the highest number of UCNEs was denoted as ‘major ortholog’, the other(s) as ‘minor ortholog(s)’(occasionally, there were more than two annotated orthologs). The classification was then based on three numbers. Let *a* be the number of UCNEs retained only by the major ortholog, *b* the number of UCNEs retained by both the major and minor orthologs and *c* the number of UCNEs retained only by the minor ortholog(s). Then a case was classified as ‘winner-takes-all’ if *a/*(*a*+ *b* + *c*) *>* 0.8, as ‘reciprocal retention’ if *c/*(*a* + *b* + *c*) *>* 0.2, and as ‘concordant retention’ in all other cases.

#### 2.4.2 Quantifying the biased retention of intronic/UTR UCNEs in fish orthologous genes

To quantitatively demonstrate the biased retention of intronic/UTR UCNEs in fish orthologs of a given species we determined a ‘winner score’ for each case. This was done as follows: first, we defined a conservation score *c_ij_* for each human UCNE *i* in each fish ortholog *j*. This score is equal to the bitscore of the optimal alignment, if an ortholog with a corresponding E-value ⩽ 10^−4^ exists and zero otherwise. The total amount of conserved UCNEs for the human reference gene is then determined as follows:

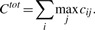


The amount of conserved UCNEs in the major orthologous gene is

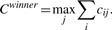


The winner score is the percentage of UCNEs retained by the major gene:

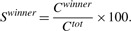

Note that we considered only the cases with two or more fish orthologs where three or more intronic/UTR UCNEs are retained in the major ortholog.

To compare the histogram of the winner scores with the expected distribution from a random retention model, we computed the mean histogram frequencies from 500 shuffled datasets. The shuffling was done by randomly permuting the assignment of conservation scores to orthologous genes for one human UCNE at a time.

### 2.5 Analysis of UCNE clusters

#### 2.5.1 Definition and gene annotation of human UCNE clusters

UCNEs that occur in arrays at orthologous chromosomal locations in the human (hg19) and chicken (galGal3) genomes were grouped into clusters. An array of UCNEs forms a cluster if (i) all UCNEs occur on the same chromosome in the corresponding genome and (ii) any two neighboring UCNEs are separated by ⩽ 0.5 Mb in both human and chicken genomes.

For each cluster, we recorded the names of the human genes associated with the cluster. A gene is considered associated with a cluster, if it contains an intronic/UTR UCNE or if it is adjacent to an intergenic UCNE from the cluster. Only protein-coding genes with annotated coding regions and not marked as pseudogenes were considered.

#### 2.5.2 Identification of orthologous syntenic subclusters of UCNEs in a fish genome

For each human UCNE cluster we identified orthologous syntenic subclusters of UCNEs in each fish genome. An orthologous syntenic subcluster is a set of UCNE orthologs that occur as a cluster on the same chromosome or scaffold in the fish genome, such that any two neighboring UCNEs are separated by ⩽0.5 Mb. From the set of orthologous syntenic subclusters we determined the one that has retained the highest number of UCNEs after whole-genome duplication and we refer to it as the ‘major orthologous subcluster’. The rest of the orthologous syntenic subclusters are referred to as ‘minor orthologous subclusters’.

Due to uncertainties or errors in the fish genome assembly, a natively syntenic region may be split over two or more discontinuous regions. To overcome this problem, we manually checked the minor orthologous subclusters and merged minor subclusters with the major orthologous subcluster under the following conditions: (i) the minor subcluster is located on a genomic region that is not assigned to any chromosome; (ii) the minor subcluster is surrounded by gaps on both sides according to the annotation provided by the UCSC Genome Browser and (iii) the two subclusters map to non-overlapping regions of the human UCNE cluster.

For each human gene that is associated with a UCNE cluster we extracted a list of corresponding othologous genes in the five fish genomes as described in Section 2.4.1. Using this list we identified fish orthologous genes that are associated with each orthologous syntenic subcluster. In a fish genome, a gene is considered associated with a UCNE subcluster if it contains an intronic/UTR UCNE or if it is located at a distance ⩽0.1 Mb from the nearest conserved UCNE of the subcluster. (Repeating the whole analysis with a distance threshold of 0.5 Mb produced virtually identical results).

Retention patterns of the major and minor orthologous syntenic subclusters were classified as described for genes in Section 2.4.1.

### 2.6 Evolutionary simulation protocol

We first defined a cluster of *N* UCNEs, all located upstream of a target gene. Then we randomly selected a fraction *q* of interacting pairs. After duplications of the clusters, we randomly applied two types of mutational events to one of the clusters at a time, synteny breaks or single UCNE knockouts. Mutations were accepted if at least one copy of each interacting pair stayed connected to the target gene. The evolutionary process was continued until no further mutations were possible. Simulations for a given parameter combination *N*, *q* were repeated 10 000 times.

## 3 RESULTS

### 3.1 Analysis of UCNE-enriched genes

We first focused on intronic and UTR-associated UCNEs of putative target genes that have been retained in two copies in one of the following five completely sequenced fish species: zebrafish, stickleback, medaka, *Tetraodon* and *Fugu*. Although this approach may be criticized for looking only at parts of a natural biological unit, namely a GRB, it offers other advantages. Most importantly, we can circumvent many difficulties related to synteny breaks and assembly errors, as annotated orthologs of human genes are free of either type of discontinuity.

To compile candidate cases, we selected 2220 UCNEs that overlapped with non-coding parts of human genes (intronic/UTR UCNEs). We ranked the human genes by the number of UCNEs they contain and selected those where we could find at least two orthologous genes and at least three conserved UCNEs in one or several fish genomes. Since many of the UCNE-enriched genes have in fact been retained in two copies after WGD, we were able to compile many test cases for our study.

**Fig. 2.**
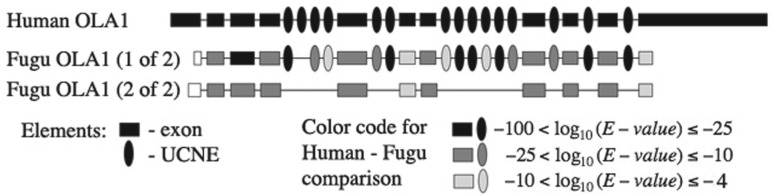
Schematic representation of a typical winner-takes-all example: one of the two orthologs of the OLA1 gene in Fugu retains many UCNEs, while the other ortholog retains none (introns are not drawn to scale)

To assess the fate of UCNEs in fish orthologous genes, we first identified sequences orthologous to UCNEs (Section 2). We then determined the number of conserved UCNEs in each fish ortholog and declared the fish ortholog with the highest number ‘major ortholog’, and the other one ‘minor ortholog’. Based on the numbers of UCNEs conserved only in the major, the minor or in both orthologs, respectively, we classified the retention patterns as ‘winner-takes-all’, ‘reciprocal retention’ or ‘concordant retention’. The ‘winner-takes-all’ retention pattern denotes that one fish gene retains many UCNEs whereas the other one loses all (or most—see Section 2). A typical example is shown in Figure 2. ‘Concordant retention’ denotes that the same UCNEs are retained in both copies of the fish orthologs. ‘Reciprocal retention’ means that different UCNEs are retained in the fish orthologs. Surprisingly, the majority of the genes exhibited the ‘winner-takes-all’ pattern (Table 1, Supplementary Fig. S1, for full results see Supplementary Table S2).

**Table 1.** Classification of retention patterns of intronic/UTR UCNEs in fish orthologs for the top UCNE-enriched genes

Gene	#UCNEs	*Fugu*	Medaka	Stickleback	*Tetraodon*	Zebrafish	Classification
NPAS3	53	6–0–0	n/a^2^	n/a^1^	1–0–1	n/a^2^	win: 1 rec: 1
DACH1	39	22–1–0	21–2–0	16–3–0	20–1–0	18–6–0	win: 4 conc:1
FOXP2	38	29–2–0	n/a^2^	n/a^2^	27–1–1	n/a^2^	winner: 2
EBF3	38	n/a^2^	27–1–0	24–4–1	28–0–0	31–0–0	winner: 4
FOXP1	38	25–0–0	19–0–0	22–0–0	24–0–0	24–0–0	winner: 5
AUTS2	34	n/a^2^	n/a^2^	n/a^2^	n/a^2^	15–4–0	concord: 1
ZEB2	27	22–0–0	15–0–0	6–1–2	19–0–0	8–5–2	w:3 r:1 c:1
ZFPM2	25	n/a^2^	n/a^2^	n/a^2^	n/a^2^	14–7–0	concord: 1
SOX6	22	14–0–0	13–2–0	14–1–0	12–0–0	n/a^2^	winner: 4
ESRRG	22	7–0–0	7–0–0	8–0–0	5–0–0	17–0–0	winner: 5
EBF1	21	2–1–2	4–0–2	3–2–0	3–1–0	5–5–0	rec:2 conc:3
PBX3	21	n/a^2^	n/a^2^	n/a^2^	n/a^2^	16–2–0	winner: 1
MEIS2	18	n/a^2^	15–0–0	13–3–0	14–1–0	7–3–0	win:3 conc:1
OLA1	16	14–0–0	13–1–0	14–0–0	11–0–0	n/a^2^	winner: 4
EHBP1	15	9–0–0	n/a^2^	n/a^2^	7–0–0	n/a^2^	winner: 2
DACH2	12	n/a^3^	n/a^1^	n/a^1^	n/a^3^	9–0–0	winner: 1
MEIS1	12	8–0–0	n/a^1^	n/a^1^	n/a^2^	9–0–0	winner: 2
NBEA	12	8–0–1	8–0–1	8–0–1	7–0–0	5–0–0	winner: 5
POLA1	12	n/a^2^	n/a^2^	n/a^2^	5–0–0	n/a^2^	winner: 1
SATB1	10	5–0–0	11–0–0	7–0–0	5–0–0	7–0–0	winner: 5

An *a*—*b*—*c* pattern stands for: *a*—number of UCNEs in the ‘major ortholog’ only, *b*—number of UCNEs in both orthologs, *c*—number of UCNEs in the ‘minor ortholog’ only. The classification column denotes the number of cases where the corresponding pattern is observed. *Notation*: n/a^1^—no orthologous gene present in the corresponding fish; n/a^2^only one ortholog present; n/a^3^—no UCNEs retained in the fish orthologous genes.

**Fig. 3.**
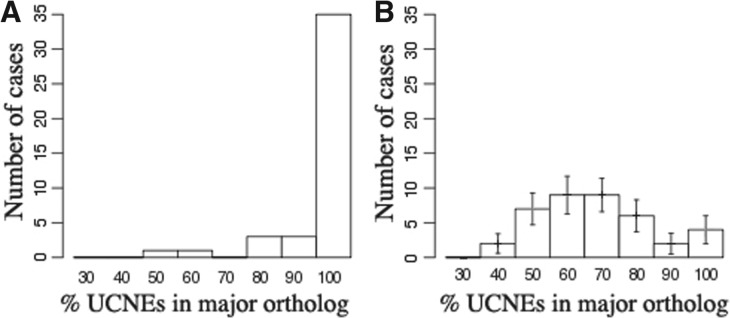
(**A**) Distribution of the amount of conserved UCNEs retained by the ‘major’ orthologous gene in Zebrafish; (**B**) Expected distribution from a random retention model based on shuffled data. Error bars represent the standard deviation computed from 500 simulations

To provide statistical support for our claim, we computed for each test case the amount of conserved UCNEs retained by the major ortholog, as a percentage of the total amount of conserved UCNEs in the two orthologs. In this analysis we considered also the length and alignment scores of the conserved UCNEs (see Section 2). The distribution of this statistic is shown in Figure 3A. We note that in 81% of the cases, one gene takes 100% of the UCNEs.

We then repeated the same analysis with permuted data reflecting a random retention model. In this case, only 9% of all genes retain 100%. Taking into account the error bars in Figure 3B, the difference is highly significant. In conclusion, we interpret the preponderance of the winner-takes-all pattern as strong evidence for a cooperative mechanism of UCNE function.

### 3.2 Analysis of UCNE-clusters

To better understand the reasons for the ‘winner-takes-all’ retention pattern, we extended the analysis to complete regulatory blocks operationally defined as clusters of UCNEs. We defined clusters as syntenic arrays with a maximal distance of 0.5 Mb between adjacent UCNEs in both the chicken and human genome. We then ranked the clusters identified in this way by the number of UCNEs and focused on the top 25 clusters (Table 2) (we assume that these clusters correspond to GRBs). Then we tried to identify ortholologous regions in the target fish genomes. This task is more difficult with GRBs than with individual genes as we have to assume that synteny breaks may have occurred in at least one of the clusters.

**Table 2.** Classification of retention patterns of UCNE clusters in fish genomes for the top 25 clusters

#UCNEs	Associated genes	*Fugu*	Medaka	Stickleback	*Tetraodon*	Zebrafish	Classification
134	**ACVR2A**; ARHGAP15; GTDC1; **ZEB2**	67–0–0	47–0–0	36–1–3	56–0–0	24–6–15	winner:4, recipr:1
96	CCNE1; **TSHZ3**; **ZNF507**; **ZNF536**	37–11–4	31–11–4	39–15–3	13–8–18	59–4–0	winner: 1, concord: 3, recipr:1
96	**EBF3**; GLRX3; MGMT	48–0–0	48–0–0	45–2–0	48–0–0	34–2–26	winner: 4, recipr:1
92	**BCL11A**; **CCDC85A**; **FANCL**; PAPOLG; **VRK2**	19–6–1	24–7–0	23–6–1	18–4–1	47–3–0	winner: 1, concord: 4
83	**FOXP2**; MDFIC; **TFEC**	57–1–0	42–0–0	59–0–0	53–0–1	43–0–24	winner: 4, recipr:1
79	**HNF4G**; **PEX2**; **ZFHX4**	36–0–0	35–0–0	37–0–0	30–0–0	58–0–0	winner: 5
73	**DACH1**; MZT1	35–1–0	32–1–0	29–1–0	34–1–0	29–6–0	winner: 5
72	**ESRRG**; RRP15; SPATA17; TGFB2; **USH2A**	18–0–0	19–0–0	22–1–1	10–0–0	49–0–1	winner: 5
71	AKAP6; EGLN3; NPAS3; SPTSSA	7–0–2	9–0–0	1–0–0	6–0–0	39–0–0	winner: 4, recipr:1
67	**ATPBD4**; C15orf41; **MEIS2**	51–0–0	46–2–0	46–4–1	41–0–0	25–3–0	winner: 5
67	ANKRD32; **FAM172A**; **MCTP1**; **NR2F1**	36–0–0	34–0–0	39–0–0	32–0–0	40–0–0	winner: 5
60	**AKTIP**; BRD7; **CHD9**; **CYLD**; **FTO**; IRX3; **IRX5**; **IRX6**; **MMP2**; NKD1; NOD2; RBL2; **RPGRIP1L**; SALL1; SNX20; TOX3	34–3–0	37–0–0	41–0–0	22–0–8	34–3–0	winner: 4, recipr:1
60	**ADK**; C10orf11; **COMTD1**; DUPD1; **DUSP13**; **KAT6B**; **KCNMA1**; SAMD8; **VDAC2**; **ZNF503**	15–22–8	17–20–6	16–22–8	16–22–4	40–0–4	winner: 1, concord: 2, recipr:2
59	**TSHZ1**; ZADH2; ZNF407; ZNF516	12–12–6	14–11–2	11–11–6	9–8–12	28–0–0	winner: 1, concord: 1, recipr:3
57	**MCTP2**; **NR2F2**	23–0–2	23–1–0	26–1–0	24–0–0	35–0–0	winner: 5
49	**FOXP1**; **MITF**	30–0–0	23–0–0	28–0–0	30–0–0	30–0–0	winner: 5
45	C1D; ETAA1; MEIS1; PNO1; PPP3R1; SPRED2; WDR92	22–0–0	14–0–0	/	21–0–0	25–0–0	winner: 4
44	MRPS9; **POU3F3**; TMEM182	9–0–0	17–1–0	2–0–2	3–0–0	23–1–0	winner: 4, recipr:1
44	**FIGN**; **GRB14**; **KCNH7**	8–13–3	10–9–4	13–10–3	10–11–3	17–2–0	winner: 1, concord: 4
43	**BNC2**; **CNTLN**	24–0–0	26–0–0	25–0–0	24–0–0	17–0–0	winner: 5
42	**FBXL4**; **KLHL32**; **MMS22L**; **POU3F2**	8–1–0	8–0–0	8–1–0	7–1–0	9–0–0	winner: 5
42	**FAM125B**; **GAPVD1**; MAPKAP1; **PBX3**	32–0–0	34–0–0	35–0–0	30–0–0	27–1–3	winner: 5
41	C8orf83; **RUNX1T1**	13–0–0	11–0–0	12–0–0	9–0–0	17–0–0	winner: 5
40	**LMO4**; PKN2	16–1–0	15–0–0	17–1–0	14–0–0	18–3–0	winner: 5
40	**KCTD1**; **PSMA8**; **SS18**; TAF4B; **ZNF521**	21–0–0	19–0–0	19–0–0	15–0–0	5–0–1	winner: 5

The potential target genes of a cluster are marked in bold. An *a*-*b*-*c* pattern stands for: *a*—number of UCNEs in the ‘major’ cluster only, *b*—number of UCNEs commonly present in the ‘major’ and ‘minor(s)’ clusters, *c*—number of UCNEs present in the ‘minor(s)’ cluster only. The classification column denotes the number of cases where the corresponding pattern is observed.

**Fig. 4.**
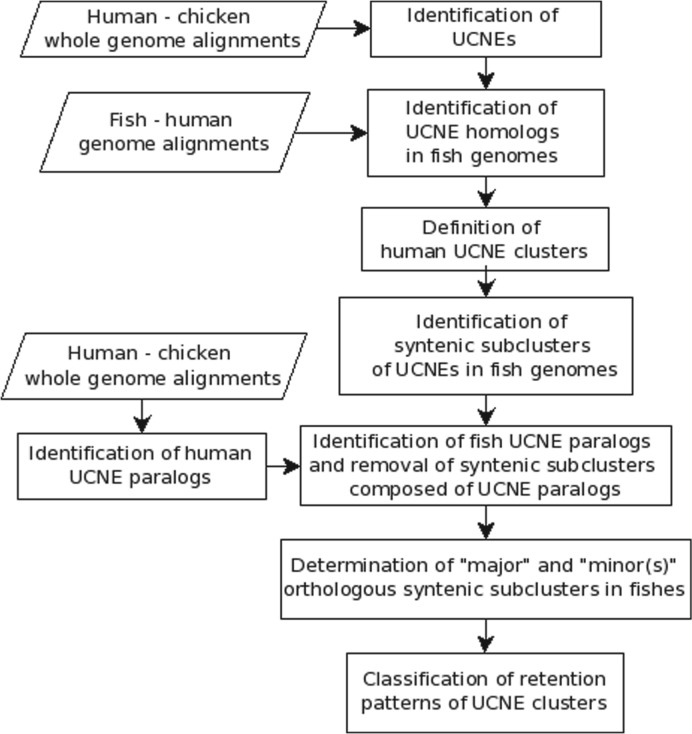
Flowchart of the analysis of UCNE clusters

Our analysis strategy is guided by the assumption that at least one cluster copy has to stay intact after WGD. Practically we proceeded as shown in Figure 4. We first searched for orthologs of the UCNEs in the fish genomes. The genomic coordinates of the UCNE orthologs identified in this way were used to define syntenic orthologous regions in the fish genomes. Typically, we found one to three syntenic regions with multiple UCNEs, occasionally also a few singletons. Next, we eliminated a few syntenic subclusters composed of ancient paralogous UCNEs resulting from earlier WGD events, some of which were described in (McEwen *et al.*, 2006). At the end, we declared the orthologous region with the highest number of orthologous UCNEs the ‘major subcluster’. All other regions were considered as ‘minor’ regions. The retention statistics was carried in the same way as for the single genes.

For each GRB, we also tried to identify potential target genes. For each gene associated to a human GRB, we checked whether its orthologs in the five fish species are UCNE-associated or not according to a co-localization criterion (see Section 2). Each gene that has at least one UCNE-associated ortholog in all five fish species is considered a potential target gene; the others are considered bystander genes.

**Fig. 5.**
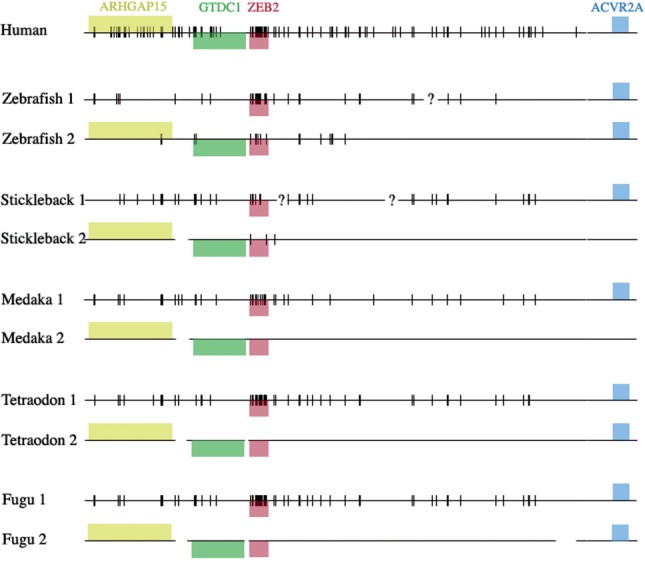
Retention patterns of genes and UCNEs of the ZEB2 cluster in five fish species. Genes are shown as colored boxes above or below the chromosomes according to their orientation. UCNEs are shown as vertical segments. Line breaks indicate discontinuities in the fish genome assemblies. Question marks indicate line breaks that may not be real. The break in the major zebrafish cluster corresponds to a local inversion potentially resulting from an assembly error

All results from the cluster analysis are summarized in Table 2 and Supplementary Figure S2 (for full results see Supplementary Table S3). Details for one example (ZEB2) are presented in Figure 5. Overall, the winner-takes-all trend is confirmed. However, we see a larger number of imperfect reciprocal retention patterns. We also were surprised to see a striking difference between the retention patterns of the same GRB in different fish species. For instance, the ZEB2 cluster displays a classical winner-takes-all pattern in all species except zebrafish, where we observe reciprocal retention.

### 3.3 Validation by evolutionary simulations

To better understand the quantitative relationship between the connectivity of a *cis-*regulatory network and the resulting retention patterns, we carried out evolutionary simulations with artificial GRBs consisting of *N* UCNEs and a fraction of *q cis-*interacting pairs. We found that at least 60% connectivity (*q* ⩾0.6) is required in order to achieve *>*75% winner-takes-all cases. Surprisingly, this relationship appears to be independent of the cluster size *N*. Although these simulations may be criticized for being over-simplified (notably they ignore potentially deleterious effects of gain-of-function mutations) they make clear that the winner-takes-all pattern is not an obvious evolutionary outcome. Within the explanatory framework we propose, the high incidence of the winner-takes-all pattern can only be explained by a high degree of connectivity of the UCNE interaction network.

## 4 DISCUSSION

In this work, we have addressed the question whether UCNEs of a GRB act independently of one another, or in a cooperative manner. Using a special setting of genomic context analysis, we presented evidence in support of the cooperative mode of action. This cooperativity can be viewed as a second layer of combinatorial gene regulation. Traditionally, this concept has been applied to transcription factor binding sites within a gene regulatory region. It has been argued that a great variety of different response behaviors could be achieved through different combinations of relatively few different transcription factors-binding sites. We propose that this kind of combinatorics extends to the next higher level of gene regulatory units, namely to GRBs composed of UCNEs.

The cooperative model of UCNE action has implications for experimental strategies to elucidate their function. At first sight the model seems to be in conflict with experiments showing that single UCNEs can drive tissue-specific gene expression in mouse or zebrafish embryos. Our findings could be reconciliated with this fact by assuming that UCNEs have an intrinsic gene activating capability with loose tissue specificity dependent on interactions with neighboring UCNEs to achieve the higher degree of regulatory precision needed for *in vivo* function. Regardless of the precise reasons, the cooperative model suggests that reporter gene assays in mouse embryos with single UCNEs cannot reveal the true function of these elements (though they may be effective in distinguishing tissue-specific enhancers from other types of UCNEs). In vein with this, our cooperative model gets support from *in vivo* deletion experiments, where combinations of conserved non-coding elements were knocked out in their native genomic context (Montavon *et al.*, 2011). In this study, complex non-additive effects of combinations of mutations were observed, and physical interactions between mutated elements could be demonstrated by chromosome conformation capture technology.

One of the open questions arising from our work is why target genes that have lost all UCNEs are nevertheless retained in extant fish genomes. One possible explanation is that they underwent neofunctionalization in the protein-coding part or regulatory regions. This hypothesis could potentially be tested by comparative genomics methods, and thus constitutes one of our agenda item for future work. We note in this context that many of the *bona fide* target genes of GRBs have paralogs that were retained after two earlier WGD events that have occurred in vertebrate evolution (e.g. Foxp1–4).

The most exciting aspect of our work is that it suggests an explanation for ultraconservation. In fact, a dense cooperativity network interconnecting many UCNEs could be responsible for both their high conservation and strong clustering on the genome. If one element interacts with many others then each interaction will add constraints on the base sequence. Similar ideas have been raised in the context of experimental studies of physical interactions between non-coding elements using chromosome conformation capture technology (Robyr *et al.*, 2011). The principle that many interactions imply high conservation is well accepted for proteins. For instance, the ultraconservation of histone proteins is commonly explained by the fact that these proteins have to properly interact with hundreds of other nuclear proteins. Hence, if ultraconservation is the consequence of interactions with neighboring elements, it follows that UCNEs can only exist in the vicinities of other UCNEs. If this view is correct then GRBs were created by a concerted evolutionary process during which the degree of conservation of individual elements has increased proportionally to the number of elements within the block. The few UCNEs which occur outside clusters may be splicing regulators or RNA genes rather than *cis*-acting regulatory elements. Their high degree of sequence conservation may also be due to many molecular interactions but not *cis*-interactions. Hence, the genes encoding their *trans*-interaction partners can be spread all over the genome.

*Funding*: S.D. was supported by grant PDFM33-120719 from the Swiss National Science Foundation.

*Conflict of Interest*: none declared.
